# Pathology and Practice of Medicine

**Published:** 1861-05

**Authors:** Richard J. Dunglison

**Affiliations:** One of the Physicians to the Pennsylvania Institution for the Instruction of the Blind; Honorary Local Secretary to the Sydenham Society of London, etc.


### PATHOLOGY AND PRACTICE OF MEDICINE.

BY RICHARD J. DUNGLISON, M.D.,

ONE OF THE PHYSICIANS TO THE PENNSYLVANIA INSTITUTION FOR THE INSTRUCTION OF THE BLIND; HONORARY LOCAL SECRETARY TO THE SYDENHAM SOCIETY OF LONDON, ETC.

I.—*Clinical Researches into Morbid Pigmentary Changes in the Complexion.* By THOMAS LAYCOCK, M.D., Professor of the Practice of Medicine, etc., in the University of Edinburgh. (British and Foreign Med.-Chir. Review, January, 1861, p. 185.)

Dr. Laycock, in consideration of the diagnostic importance of color-characters, and the fact that, in spite of their varied applications to practical uses, they are so imperfectly understood, devotes a paper to the clinical meaning and pathology of morbid pigment—deposits and pigmentary changes in the complexion. Clearer views as to the diagnosis, pathology, and treatment of certain related groups of constitutional diseases may be deduced as well from the absence of pigment-deposit as its presence.

The conclusions which Dr. Laycock proposes to illustrate are as follows:—

1. That besides blue and green, of rare occurrence, there are two common well-marked and distinct forms of morbid discoloration due to pigment-deposit, the *yellow* or sallow, and the black or *swarthy.*

2. That both yellow and swarthy discoloration of the skin will occur from the action of local irritants—as heat, light, cutaneous parasitic fungi, blisters, sinapisms, and the like, or in the progress of various diseases of the skin and its appendages.

3. That the absence of pigment (leucopathia) as well as its deposit, may be caused by inflammatory and other diseases of the skin, affecting its chromatogenous function.

4. That morbid states of the cerebro spinal centres will influence the deposit or non-deposit of pigment.

5. That morbid states of the genito-urinary organs in both sexes, acting probably through the nervous system, will determine the election of locality of pigment-deposit, according to the same law by which the development of sexual hair and pigment is regulated.

6. That structural disease of the abdominal viscera and peritoneum also exercise an influence through the nervous system upon the local deposit of pigment in the skin.

7. That in disease of the supra-renal capsules, the bronzing of the skin, whether swarthy or yellow, is partly nervous, and due to the direct or indirect influence of the capsules or the kidneys and nervous system; partly hæmic, and is so far due to the morbid influence of "dyscrasic" blood.

8. That pigmentary changes in the skin of both whites and blacks may be the *result* of morbid causes, and yet may remain after the operation of the causes has ceased, and assume a physiological character.

9. That although local morbid pigmentation of the skin may occur exclusively from local causes, or the influence of the nervous system, in the majority of cases there is a morbid condition of the blood.

10. That the morbid conditions of the blood associated most commonly with pigmentary changes are characterized by those changes in the blood corpuscles (leukæmia, leucocytosis) which are observed in cachectic states of a constitutional character, (pregnancy, chlorosis, tertiary syphilis, chronic rheumatism, cancer, etc.,) or which are intimately connected with "dyscrasic," visceral, or glandular diseases (of the spleen, supra-renal capsules, lymphatic glands.)

11. That the tendency to discoloration increases (*cæteris paribus*) with age after a certain period of life.

12. That the morbid pigment-deposits proper, as distinguished from masses of altered blood corpuscles, are carbonaceous excretions, and are often vicarious with the suspension or imperfect elimination of other carbonaceous excretions, as the carbonic and lactic acids, and the pigment constituents of both the urine and bile; and are consequently associated with morbid states of assimilation, as well as of elimination, (through the skin, lungs, liver, kidneys.)

13. That among the morbid states of assimilation, the rheumatic and gouty are specially to be classed, as well as those coincident with anæmia.

Dr. Laycock enters as well into the literature of the subject as into the pathology; but as this interesting paper is but the first portion of his article on Cutaneous Discolorations, we forbear at present from doing more than referring to the general outlines and objects of the task which he has mapped out for himself.

II.—*Analysis of Fifty-Two Cases of Epilepsy.* Observed by DR. EDWARD H. SIEVEKING. Described by the author before the Royal Medical and Chirurgical Society, Feb. 12, 1861. (London Lancet, Feb. 23.)

This paper is the second contribution made by Dr. Sieveking to the statistical history of Epilepsy, and embraces the same number of cases as were subjected to analysis in the summary presented by him to the society in 1857. Only such points are detailed as are supported by satisfactory evidence of their accuracy.

*Sex.*—Twenty-three were females, or 44·2 per cent.; 29 were males, or 55·8 per cent. Taking the two series together, the ratio of females to males was as 45·2 to 54·8.

*Age.*—Based on the time at which the epilepsy first exhibited itself:—

| | | | |
|---|---|---|---|
| Under 10 years . . . . | 12 cases, | 8 males, | 4 females. |
| From 11 to 20 years . . | 25 " | 11 " | 14 " |
| " 21 to 30 " . . . | 7 " | 5 " | 2 " |
| " 31 to 40 " . . . | 3 " | 3 " | 0 " |
| " 41 to 50 " . . . | 2 " | 1 " | 1 " |
| Above 50 . . . . . | 3 " | 1 " | 2 " |
| | 52 | 29 | 23 |

*Causes.*—Hereditary taint was traceable in 14 cases, but in 8 only of these was there evidence of epilepsy having occurred in a near relative of the patient. Various exciting causes were traced in 37 cases.

*Premonitory symptoms.*—Comprising under the term "aura" all symptoms indicative of a near approach of a paroxysm, the author found it in 21 cases, or 40 per cent., or less frequently than in the first series, where 52 per cent. of the cases exhibited premonitory symptoms.

*Headache* was constant or frequent in 9 cases, or 17·3 per cent.; occurred after the seizures only in 12 cases, or 23·0 per cent.; and only immediately before or after the fits in 3 cases, or 5·7 per cent.

*Biting the tongue*, in the first series, was met with only in 32·7 per cent.; in the present series, 28 patients, or 53·8 per cent., exhibited this feature. Females are not less prone to inflict this injury upon themselves than males, for of the 24 cases in which the tongue had not been bitten, 16 were males and 8 females.

*Urine.*—The author has found various derangements in the urine associated with epilepsy, but none of them had been constant.

*Treatment.*—The author advocates no specific treatment, but recommends perseverance in a combination of moral, regiminal, and medical means. He considers that he can lay claim to eight cures, fifteen were decidedly benefited, while the remainder were either uninfluenced by treatment, or did not continue under observation for a sufficient period to justify any positive statement as to the result.

III.—*A Case of "Bloody Sweat."* By THOMAS K. CHAMBERS, M.D., Physician to St. Mary's Hospital. (London Lancet, March 2, 1861.)

Dr. Chambers devotes a whole Clinical Lecture at St. Mary's Hospital to the history of a case of vicarious hemorrhage, a patient "whose bleeding brow and blood-streaked cheeks," according to the lecturer, "painfully recalled the attempts of mediæval art to realize the agonies of Gethsemane, or the thorn-crown worn, in Pilate's judgment-hall for our sake."

Up to the age of twenty-three, the catamenia had appeared but once or twice. At this time commenced in the skin of the face peculiar symptoms, which, continuing to the present time, may be described as follows: she feels first a peculiar soreness and tenderness of an isolated spot, which enables her to predict that in the course of a few hours an eruption is going to commence. The first appearance of this is an erythematous blush, sometimes slightly raised above the surrounding surface, but not so much as in erysipelas. After a short time, a scattered crop of fine vesicles like sudamina appear, mixed with a fine serous dew not covered by any pellicle. This never lasts long enough to form colorless drops, for quickly it becomes blood-stained, and the little points of blood are seen oozing out, sometimes so slowly as to dry and form a scab, sometimes collecting into great thick gouts, and trickling in a ghastly way down her face.

The eruption runs through its stages most quickly when she is asleep in bed. The bleeding is increased and prolonged, if the part be rubbed or washed; but if left alone to dry in a scab, it stops in a week or ten days, to be succeeded before it is quite recovered by a similar eruption in another place. Sometimes the cutaneous phenomena were replaced by bleeding from the nose, sometimes by vomiting of blood, but never by hemorrhage from either lungs or bowels.

She finally recovered, after leeches had been applied to the spot at which the bleeding was expected; but was admitted into St. Mary's Hospital as a mild case of erysipelas of the face. But when it began to bleed, its nomenclature, pathology, and treatment, puzzled Dr. Chambers not a little.

But here, in addition to the facial hemorrhage, the legs, thighs, forearms, and chest exhibited at different times similar appearances. With all these derivations, she appears healthy in other respects, and does not seem to be weaker in muscular power than on admission. When the uterine function was temporarily re-established, there was no immediate lessening of the cutaneous hemorrhage; but the woman is now so much improved as to have become an out-patient.

In commenting on this interesting case, Dr. Chambers refers to the rarity of this form of vicarious hemorrhage, and to the difficulty of finding any records of a similar kind. The cases which he has been able to collect may be summed up in a few words; they begin with Haller's account of eighteen cases, and but few have been recorded since. The literature of the subject is scanty, simply because the number of patients is scanty.*

From this case and the others recorded previously, Dr. Chambers arrives at the following conclusions:—

1. Cutaneous menstruation occurs in robust and healthy-looking, rather than in anæmic, persons. It appears in fact to be a plethora.

2. It may occur without any detectible disease of the parts of generation.

3. It may also be caused by disease in those parts.

4. The catamenia are not necessarily entirely diverted from their usual channel.

5. Periodical cutaneous hemorrhage is not necessarily connected with the catamenia at all, for Van Swieten quotes the case of a man.

6. It is not always strictly periodical.

7. Suppressio mensium may go on for many years without any tendency to vicarious menstruation.

8. Cutaneous menstruation usually appears in the shape of a slightly swelled erythema, painful and tender, on which vesicles form; the vesicles quickly burst, and there is poured forth a sero-sanguinolent exudation, which then becomes bloody, and continues to exude like a bloody sweat for various times, from four days to a fortnight. No scar is left, but the skin is slightly discolored for some time afterwards in the parts affected.

9. Perhaps we ought not to call it a disease of the womb any more than a disease of the skin.

10. Blood-letting is the most efficacious treatment; and it is most efficacious in the form in which it is known to produce its most powerful physiological effect, namely, in small repeated doses, and as close as possible to the seat of action, that is, on the spots affected. The measure of the good effect of the blood-letting is the relief experienced by the patient without loss of strength.

11. No other remedies are as yet known to be of any advantage.

---

* A case of vicarious hemorrhage, presenting some of the phenomena described in Dr. Chambers's case, is referred to by Professor Dickson, as occurring in his practice.—(*Elements of Medicine*, 2d ed., p. 375. Philad. 1859 )

IV.—*Hypochondriac Delirium a Forerunner of Paralysis.* (Journal de Médecine et Chirurgie.)

It not unfrequently happening that the symptoms by which general paralysis is ushered in escape notice, it is all-important to recognize their existence if possible. Among these M. Baillarger indicates, as deserving of attention, hypochondriac delirium. He asserts that it is a serious presumption of the future occurrence of general paralysis, and adds one element to the prognosis of the disease.

Bayle's interesting and valuable researches have made it abundantly plain, that delusions marked by much elation, or the *manie des grandeurs*, are in many instances the premonitory signs of paralytic dementia. If it is really the case that the delusion of fancied supereminence is of such great prognostic value in monomania, why should not the same be true also of the hypochondriac delirium of melancholics?

M. Billod, chief physician of the Asylum of Sainte-Gemmes-sur-Loire, afterwards endeavored to show that M. Baillarger's remarks on hypochondriac delirium, as a forerunner or a *pathognomonie* of general paralysis, are equally applicable to all forms of melancholic delusion whatever their nature; for instance, to fancied persecution, and that the important fact adduced by the professor of La Salpetrière refers more to the variety of melancholia attended with stupor, than to the peculiar nature of the delusions by which it is accompanied. M. Billod agrees with M. Baillarger and most authors, that general paralysis may be characterized as well by melancholy and depression, as by elation with large delusions; and he has even found both forms of mental aberration combining in the same subject to induce a mixed mental condition, in which notions of ideal wealth and grandeur were associated with the chimerical dread of persecution.

M. Brierre de Boismont has sought for the first indications of general paralysis in the perversion of the moral and affective faculties. Of all moral perversions, that which has especially struck M. Brierre de Boismont is kleptomania; in the second place, he considers as highly suggestive the perpetration of shamefully licentious acts, in contrast with previously correct habits of life.

V.—*Case of Recurrent Anæsthesia of almost the entire Surface of the Body, accompanied by Partial Loss of Motor Power in all the Limbs, traceable to the Effects upon the Spinal Nerves of Effused Products within the Spinal Canal.* By JOHN W. OGLE, M.D., Assistant Physician to St. George's Hospital. (Reprinted from vol. xliii. of the Medico-Chirurgical Transactions, published by the Royal Medical and Chirurgical Society of London, 1860.)

The great infrequency of the post-mortem appearances met with in this case, the character, cause, and mode of access, etc., of the various symptoms, and the close and intelligible dependence of the symptoms upon the lesion, as determined after death, give a special interest to this case described by Dr. Ogle.

*Symptoms.*—Before his admission into the hospital, he had suffered every winter for about six years from numbess and the peculiar sensation usually termed "pins and needles" in his lower limbs, and when brought to the notice of the hospital presented the following interference with motor power and general sensibility of the integuments. The leg, as far up as the middle of the thigh, both arms, at all parts below the elbows, both cheeks, and both sides of the nose, were almost insensible to pinching or pricking. The muscular power was much weakened, both in the arms and legs.

After appropriate treatment, which included blisters, diuretics, mercurials, and nux vomica, he enjoyed for a time entire immunity; then was subject to slight relapses every year, but only in the winter time; then had a relapse so severe as to necessitate discontinuance of his work; and, after partial recovery, slight attacks, the upper limbs only (and chiefly the right one) being affected; and finally, after several more slight invasions, was attacked by his last illness, which differed materially from all previous attacks. When last admitted, he was rambling and soporose, and his mental condition was such that Dr. Ogle found it difficult to discover to what extent the powers of motion or the sensibility of the skin were interfered with. He died on the third day after admission, having been in a state of stupor during nearly the whole period.

*Post-mortem appearances.*—The most interesting changes observed were in the spinal column, chiefly at the lower cervical and the upper dorsal regions, the anterior and the posterior roots of the nerves being invested by very firm fibrinous material, partly of a white and partly of a pinkish color, the deposits being sufficient in some places to form a layer, obscuring and matting together the various nervous strands composing the entire nerve-root. In one place, it formed a pink-stained mass around one of the anterior roots on the right side, of the size of an ordinary pea, and having much the appearance of a neuroma.

*On microscopical examination*, Dr. Ogle found that the great part of the material investing the nerve-roots possessed all the histological appearances of ordinary fibrinous material; but in some portions there were discovered vast numbers of peculiar round and oval and oat-shaped bodies. Where the masses about the nerves were pinkish, blood-globules were found mixed with the material.

*Remarks.*—The author's view of the nature of the adventitious material referred to above is, that it was manifestly the remains of some exudative process which had during life affected the spinal membranes. This may have been of a purely local nature, and of a kind usually designated inflammation of the spinal membranes, (the masses of deposit about the nerves being, in fact, only part of some exudation, the rest, chiefly fluid, having been absorbed,) or a mere expression or manifestation, that is, of some general cachectic state such as would induce fibrinous effusions simultaneously into various organs and upon various free surfaces. The roots might thus become compressed as if a ligature were passed around them.

Dr. Ogle finds a difficulty in explaining the lengthened intervals occurring in such a chronic series of attacks. From the course taken by the symptoms, the nerves of the lower part of the cord were doubtless first affected, then those of the upper part, and finally the fifth cranial nerves. With all this morbid condition of the spinal nerves, the freedom from pain and from pathological muscular contraction, until late in the disease, can only be accounted for by the extreme slowness of the diseased action. Mental disturbance just before the termination of life no doubt corresponded with the invasion of the sub-arachnoid tissues at the base of the brain, and with the softening of the walls of the ventricles, with effusion into their cavities.

VI.—*On the Restoration of Suspended Animation in Persons Apparently Drowned.* By DR. CHRISTIAN. (London Lancet, Feb. 2, 1861.)

A communication to the Royal Medical and Chirurgical Society by Dr. Christian, Medical Officer of the Royal Humane Society, elicited considerable discussion as to the proper means to adopt in the cases referred to. It appears that two societies, the Royal Humane Society and the National Life-boat Institution, issue instructions which are not uniform either in detail or in principle. They differ on two important points—

1. As to the mode of performing artificial respiration.
2. As to the propriety of using the warm bath.

During the twelve years of Dr. Christian's service, 443 submersions had occurred; 181 of which were rescued and recovered without treatment; 165 were treated successfully at the Receiving-house; 97 were brought dead or were unsuccessfully treated. It seems that the Life-boat Institution uses Dr. Marshall Hall's "Ready Method," while the other society employs the method of Dr. Silvester.

The latter, it seems, had given Dr. Hall's method a fair trial in fifteen cases, in order to test its efficiency, but all parties interested were firmly convinced that Dr. Silvester's method was in every way superior, more manageable, less likely to injure the patient, will fill the chest with air and expel air from it more fully, and will not force the contents of the stomach upward and in the way of respiration. The directions for treating the asphyxiated at the Receiving-house, Hyde Park, may be appropriately introduced here :—

"Wipe the mouth and nostrils directly the body is taken from the water.

"Use Dr. Silvester's method; at the same time let the body be taken as quietly as possible to the Receiving-house, and place it in the bath up to the neck.

"Raise the body in twenty seconds from the water, and dash cold water against the chest.

"Pass ammonia under the nose.

"Use again Dr. Silvester's method, and the inflating apparatus, if it fail.

"Remove the body from the bath, and rub the surface with dry hot towels, perseveringly continuing the other treatment."

The author thinks inflation of the lungs, by Dr. Silvester's method or by the society's apparatus, the first remedy, and the shock of the warm bath the second; that after eight minutes' complete submersion, recovery is hopeless, and that when ten minutes elapse after being taken from the water without any effort at respiration, it is equally so.

In the discussion that ensued on the presentation of this paper, Dr. Sharpey and others participated. The former thought that many of the cases of resuscitation in which the "Ready Method" had been successful were still-born infants, some of whom would have recovered, if left alone. He thought that in many of the cases of recovery after submersion in adults, respiration commences spontaneously as soon as the patient reaches the air; not, therefore, in consequence of the means used for resuscitation, but in spite of them. He was in favor of the old method of insufflation, the emphysema occasionally produced by it being due to the quick forcing of air in too great a quantity into the lungs, and in unsuccessful cases, in which insufflation had been practiced, no emphysema had been found at the autopsy. Dr. Sharpey thought other objections to the modes suggested were untenable.

Dr. Sibson, assisted by Mr. Hunter and Mr. Holmes, had made unsuccessful experiments with Dr. Hall's method. Mr. Spencer Wells suggested that the operation for insufflation might best be performed by passing a tube through one nostril into the glottis; accoucheurs were in the habit of inflating the lungs by passing an elastic tube down the glottis. Dr. Christian had never found emphysema to result from the use of the bellows when properly regulated, and was anxious for the adoption of definite rules and instructions upon the best modes of restoration of suspended animation. It is to be hoped that the arguments and evidence on both sides will be carefully weighed, and the merits of each mode rigidly tested by a competent commission.

VII.—*The Nature and Origin of Epileptiform Convulsions caused by Profuse Bleeding, and also of those of True Epilepsy.* By ADOLF KUSSMAUL and ADOLF TENNER, M.D. Translated by EDWARD BRONNER, M.D. (A Monograph published by the New Sydenham Society of London.)

The chief results obtained from these careful researches may be concisely grouped under the following heads:—

1. The convulsions appearing in profuse hemorrhage of warm-blooded animals (including man) resemble those observed in epilepsy.

2. When the brain is suddenly deprived of its red blood, convulsions ensue of the same description as those occurring subsequent to ligature of the great arteries of the neck.

3. Epileptic convulsions are likewise brought on when the arterial blood rapidly assumes a venous color, as, for example, when a ligature is applied to the trachea.

4. It is highly probable that in these cases the attack of spasms depends upon the suddenly-interrupted nutrition of the brain. It is not caused by the altered pressure which the brain undergoes.

5. Epileptic convulsions in hemorrhage do not proceed from the spinal cord, nor from the cerebrum; their central seat is to be sought for in the excitable districts of the brain lying behind the thalami optici.

6. Anæmia of those parts of the brain situated before the crura cerebri produces unconsciousness, insensibility, and paralysis in human beings; if spasms occur with these symptoms, some excitable parts behind the thalami optici must have likewise undergone some change.

7. Anæmia of the spinal cord produces paralysis of the limbs, of the muscles of the trunk, and of respiration. When the anæmia suddenly attains its greatest intensity, then only, and even then but rarely, do slight trembling movements of the limbs precede paralysis. The sphincter ani acts analogously to the constrictor muscles of the face in anæmia of the brain, that is, it contracts spasmodically before it relaxes.

8. Convulsions from hemorrhage are neither physical nor reflective; they do not ensue

*a.* In cold-blood animals, at least not in the frog.

*b.* When the hemorrhage is slow, so that the vital power is only gradually consumed.

*c.* When the animals are very much debilitated.

*d.* When the nutrition of the spinal cord has suffered.

*e.* When large pieces of the excitable districts of the brain have been removed.

*f.* In animals subjected to etherization.

*g.* Doubtless, also, when excitable districts of the brain have undergone certain pathological alterations.

9. As suffocation brings on convulsions, and etherization averts them, it is evident that etherization and asphyxia are two different conditions.

10. The brain of warm-blooded animals can only be deprived of red blood for a short time; otherwise it loses its capability of resuming its functions when again supplied with the nutritive fluid, and the appearance of death becomes a reality. The brain of some rabbits preserved this capability for two minutes.

11. It is sometimes observed, after the arteries of the neck have been tied, that the muscles of the trunk perish and take on the rigor mortis before the action of the left heart is extinct. Hence the left heart is not always the *primum moriens* among the muscular organs.

12. Contraction and subsequent extreme dilatation of the pupils in the agonies of death is no certain sign of real death, and of the incapability of being revived, as maintained by Bouchut.

13. To cure epileptic attacks caused by anæmia, there is no better method than that of renewing the supply of red blood. The debilitating method of treating epilepsy, especially by *abstracting* blood, should almost always be rejected.

14. The quantity of blood in the cranial cavity can, by way of experiment on the living subject, be considerably increased or diminished.—Hyperæmia in the cranial cavity is caused by releasing the stoppage of circulation in the cervical arteries, (arterial congestion,) by tying the cervical veins, (venous congestion,) especially by simultaneously dividing the cervical branches of the sympathetic nerve, (venous arterial congestion,) and lastly by tying the trachea during inspiration, (venous congestion by asphyxia.)—Anæmia in the cranial cavity is produced by hemorrhage and by tying the cervical arteries, (passive anæmia,) as well as by electric excitation of the vaso-motor nerves of the head, (active anæmia.)

15. The quantity of blood contained in the cranial cavity after the application of a ligature to the arteries is greater than after hemorrhage; the anæmia, as regards small arteries, the capillaries, and the smallest veins being always present to a greater extent.

16. From the quantity of blood contained in the skull after death, it is seldom possible to draw certain conclusions with respect to the quantity contained during life. The death-struggle brings on numerous conditions altering the circulation of the blood in the skull, and even in the corpse the quantity of blood may still undergo alterations.

17. The phenomena of the incomplete epileptic attack can be explained by alterations occurring in the cerebrum only; while the phenomena of the complete attack presuppose an alteration of the whole brain.—Convulsions in epilepsy are justly styled cerebral ones, and the spinal cord probably plays only the part of a conductor, transferring the impetuses it receives from the brain to the muscles.

18. Circumscribed anatomical alterations of the brain, or alterations of protracted duration, cannot be regarded as the proximate cause of epileptic attacks, but may cause epileptic *affections* (dispose to epilepsy.)

19. Pathological anatomy cannot give any explanation as to the nature of epilepsy.—Suddenly-withheld nutrition is only one of the causes by which the brain is brought into that peculiar internal condition which is manifested in the form of an epileptic attack.—Arterial congestion of the brain does not seem to be capable of producing any other symptoms than those of paralysis, (dizziness and apoplexy.)—Venous congestion of the brain, as well as arterio-venous congestion, brings about conditions which belong more to those of apoplexy than to those of epilepsy, and are characterized by paralysis of the glottis, together with a slower respiration and slight spasmodic symptoms.

20. Marshall Hall's sphagiamus and trachelismus are not to be regarded as a source of epileptic attacks, but the latter will produce them. All theories are false which assert the epileptic attack to be derived from a sudden determination of blood, whether active, passive, or mixed.—It is probable that certain forms of epilepsy result from a spasm of the muscular coats of the cerebral arteries.

21. The epileptic affection which disposes to the attacks occupies either the whole of the brain or some districts only, and by it the brain is brought into that altered state on which the epileptic attack is based.—The medulla oblongata, as being the part whence the nerves causing the constriction of the glottis and the vaso-motor nerves take their rise, seems frequently to be the spot from which eclamptic and epileptic attacks proceed.

